# Neuropathic pain after peripheral nerve injury in rats: a model using sciatic nerve clamping

**DOI:** 10.1007/s00540-025-03541-7

**Published:** 2025-08-02

**Authors:** Michio Kumagai, Shigekazu Sugino, Toru Murakami, Hideaki Obata, Masanori Yamauchi

**Affiliations:** 1 Department of Anesthesiology and Perioperative Medicine, Tohoku University Graduate School of Medicine, Seiryo-Machi 2-1, Aoba-Ku, Sendai, Miyagi 980-8575 Japan; 2 Department of Anesthesiology, Saitama Medical University Saitama Medical Center, Kawagoe, Kamoda 1981, Saitama 350-8550 Japan

**Keywords:** Peripheral nerve injury, Trauma, Neuropathic pain, Animal model

## Abstract

Various preclinical rodent models have been established and utilized to elucidate the mechanisms underlying neuropathic pain. However, the utility of existing major models faces challenges, such as reproducibility and surgical complexity. Here, we introduce a new rat model of neuropathic pain created by simply clamping the sciatic nerve for 10 min. First, we investigated pain-related behavior in 12 male Wistar rats after surgery to clamp the sciatic nerve that induced mechanical allodynia for 21 days and thermal hyperalgesia for 7 days (2-way ANOVA compared with sham-operated rats, *p* < 0.01). We observed only small interindividual differences in response to stimuli among the rats after sciatic nerve clamp surgery. The L5 dorsal root ganglion and spinal dorsal horn of the other 14 rats were subsequently sectioned and stained for Atf3 and Iba-1 immunoreactivity, respectively. Compared with sham surgery, nerve clamping induced peripheral and central sensitization (Mann–Whitney *U* test, *p* < 0.01). This new preclinical rat model of neuropathic pain offers procedural simplicity and high reproducibility.

**Supplementary Information:**

The online version contains supplementary material available at 10.1007/s00540-025-03541-7.

Neuropathic pain arises from damage to the central or peripheral nervous system from disease or injury [1, 2]. This uncomfortable sensation is a significant healthcare issue that causes suffering and negatively impacts quality of life [3]. Neuropathic pain is often not adequately managed because the mechanisms remain poorly understood [4]. A diverse range of preclinical animal models of neuropathic pain have been established and utilized to elucidate the fundamental mechanisms underlying this condition [5].

The main conventional animal models of neuropathic pain have been developed in rodents and have advantages and disadvantages [2, 5]. In the late 1980s, Bennet and Xi et al*.* developed a rat model of peripheral neuropathy using chrome-treated sutures loosely tied around the sciatic nerve to produce a chronic constriction injury [6]. Seltzer et al*.* developed a rat model of neuropathic pain with causalgiform features using a partial nerve injury induced by tightly ligating the dorsal third or half of the sciatic nerve [7]. These two models have been reproduced in mice and reflect some features of neuropathic pain in humans [8, 9]. Peripheral neuropathy-induced pain-like behaviors were evaluated in these models; however, a common limitation and point of criticism of these models is their reproducibility [10]. The outcomes depend highly on the surgical skills of the investigators and the materials used (e.g., surgical sutures) [10]. By contrast, Kim and Chung developed a rat model of peripheral neuropathic pain using tight ligation of the L5 and L6 spinal nerves unilaterally in a stereotyped procedure [11]. Although their model is more reproducible than those previously described, the surgical procedure is complex and challenging.

Here, we developed a new rat model of neuropathic pain following sciatic nerve injury by clamping the nerve for 10 min. This new method is characterized by procedural simplicity and high reproducibility.

The present study was approved by the Tohoku University Institutional Animal Care and Use Committee (2016MdA-313) and is reported following the ARRIVE statement guidelines [12]. We used 46 male Wistar rats (8 weeks old, 250–300 g). All rats were housed for one week in our institutional animal husbandry area in groups of 3 or fewer in plastic cages on soft bedding at 23 °C under a 12-h light/dark cycle and allowed standard laboratory chow pellets and tap water ad libitum.

The rats were subsequently transported from the animal husbandry area to the laboratory. After at least 60 min of acclimatization, a longitudinal skin incision (approximately 3 cm) was made on the lateral side of the left thigh under isoflurane anesthesia. The sciatic nerve was identified by blunt dissection. A disposable hemostatic clip (3.0 mm width, Natsume-Seisakusho, Tokyo, Japan) was used to clamp the nerve at an applied pressure of 60 g/mm^2^ for 10 min, 2 mm proximal to where the sciatic nerve trifurcates into peroneal, tibial, and sural nerves (Fig. 1A). The clip was then released, and the superficial muscle and skin were closed in each layer using 4–0 braided polyglactin 910 sutures (Vicryl; Ethicon, Bridgewater, NJ, USA) and 3–0 braided nylon sutures (Surgilon; Covidien, Minneapolis, MN, USA). In the sham-operated rats, the sciatic nerve was exposed in the same manner as described above but without clamping. The incisions were closed after 10 min.

**Fig. 1:**
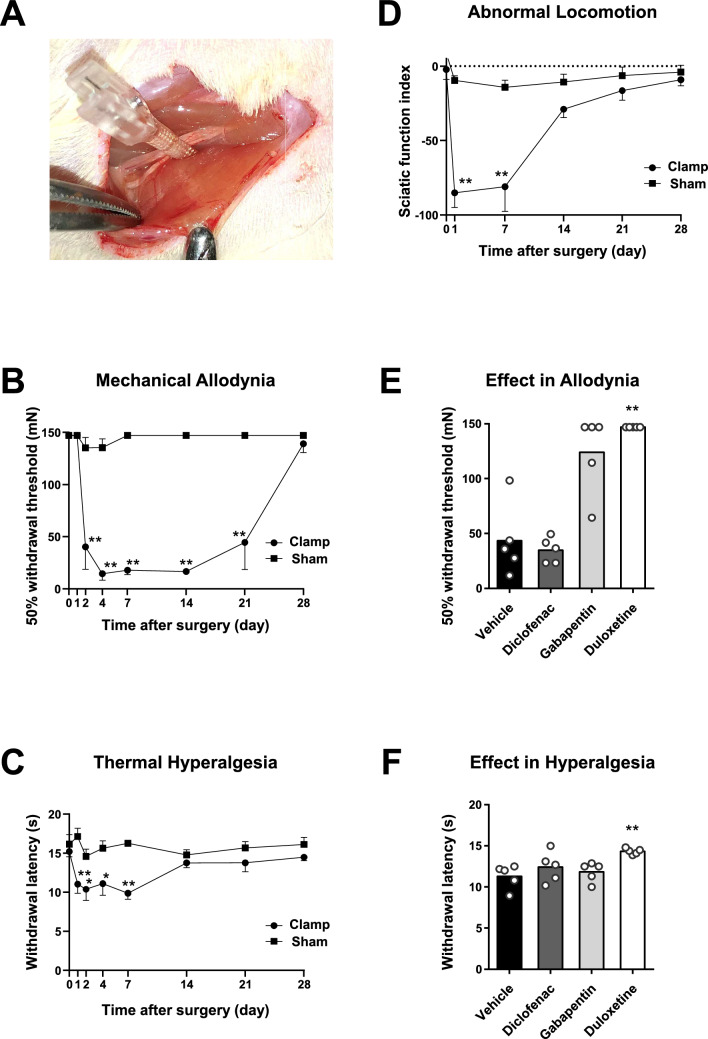
Behavioral analyses after sciatic nerve clamp surgery. **A**. Image showing clamping of the left sciatic nerve using a disposable hemostatic clip. **B**. Mechanical allodynia in the von Frey test. We assigned 12 rats to the clamp surgery group (Clamp, n = 6) or the sham surgery group (Sham, n = 6). Data are presented as the means ± standard errors of the means (SEM). Panel B shows that the mechanical allodynia persisted for 21 days after sciatic nerve clamping (2-way repeated-measures ANOVA, *p* < 0.0001; **p < 0.01 in a post hoc Sidak test vs. Sham). **C**. Thermal hyperalgesia in the Hargreaves test. Panel C shows that thermal hyperalgesia persisted for 7 days after sciatic nerve clamping (2-way repeated-measures ANOVA, *p* = 0.0008; *p < 0.05, **p < 0.01 in a post hoc Sidak test vs. Sham). **D**. Locomotion in the walking track test. Panel D shows abnormal locomotion seven days after sciatic nerve clamping (2-way repeated measures ANOVA, *p* = 0.0011; **p < 0.01 in a post hoc Sidak test vs. Sham). The sciatic functional index indicates that a value of 0 indicates normal function, and a value of − 100 indicates total impairment. **E**. Bee swarm plot representing the 50% withdrawal threshold to mechanical stimuli 30 min after administration of the three types of drugs. The bars represent the average values in the four groups: vehicle, diclofenac, gabapentin, and duloxetine (n = 5 in each group). Duloxetine attenuated the withdrawal response (Kruskal–Wallis test, *p* = 0.002; **p < 0.01 in a post hoc Dunn test vs. vehicle). **F**. Bee swarm plot representing the withdrawal latency to thermal stimuli 30 min after three types of drug administration in the same rats as in Panel E. Duloxetine attenuated the withdrawal response to mechanical stimuli (Kruskal–Wallis test *p* = 0.02; **p < 0.01 in a post hoc Dunn test vs. vehicle)

To evaluate the properties of our rat model of neuropathic pain, we performed two somatosensory behavioral experiments (von Frey and Hargreaves tests) and one motor behavioral experiment (walking track test) [13–16]. All rats were allowed to acclimate for 20 min before the measurements were made.

In the von Frey test, the withdrawal response to non-noxious punctate mechanical stimulation was determined by applying calibrated monofilaments to the mid-plantar region of the left (ipsilateral) hind paw. After a series of applications, the 50% withdrawal threshold was determined using the up–down method described by Dixon [17]. Figure 1B shows the results of the von Frey test before and on days 1, 2, 4, 7, 14, 21, and 28 after surgery. Mechanical allodynia persisted for 21 days after the sciatic nerve clamping. Allodynia returned to preoperative control levels by day 28. Notably, the interindividual differences were minimal on days 4, 7, and 14.

In the Hargreaves test, the hind paw withdrawal latency to noxious thermal stimulation was measured to the nearest 0.1 s, and a cutoff of 20 s was used. Figure 1C shows the results of the Hargreaves test before and on days 1, 2, 4, 7, 14, 21, and 28 after surgery. Thermal hyperalgesia persisted for 7 days after sciatic nerve clamping. The hyperalgesia returned to preoperative control levels by day 14. The interindividual differences were notably small, like those for mechanical allodynia.

In the walking track test, the plantar surfaces of the hind paws were painted with black ink. Each rat was placed on a sheet of white paper and guided to walk straight along a narrow alley toward a dark compartment. The sciatic functional index was calculated using the most legible footprint of each hind paw. The walking track test was performed before and on days 1, 7, 14, 21, and 28 after surgery to assess locomotor function recovery. Figure 1D shows abnormal locomotion 7 days after sciatic nerve clamping. Abnormal locomotion returned to preoperative control levels by day 28.

In a separate subset of the rats, we intraperitoneally administered diclofenac (10 mg/kg) [18], gabapentin (50 mg/kg) [19], duloxetine (10 mg/kg) [20], or normal saline as a vehicle control on postoperative day 14 after complete wound healing to examine the pharmacological effects on the model. Gabapentin and duloxetine, but not diclofenac, are first-line drugs for the treatment of neuropathic pain in humans [21]. At 30 min after drug administration, we used von Frey and Hargreaves tests to measure changes in pain-related behavior in response to the treatments. Duloxetine attenuated the responses to mechanical and thermal stimuli, as observed in humans (Fig. 1E and 1F). Gabapentin tended to attenuate the response to mechanical stimuli, but the attenuation was not significant (Fig. 1E).

To determine the molecular changes in dorsal root ganglia (DRG) induced by peripheral sensitization after clamping the sciatic nerve, 3 cross-sections of the left (ipsilateral) L5 DRG were stained immunohistochemically with anti-activating transcription factor 3 (Atf3) IgG and with 4′,6-diamidino-2-phenylindole (DAPI) 7 days after surgery. The number of Atf3-positive cells, indicating neuropathy [22, 23], was counted manually, and the ratio of positive cells to total cells was calculated (Fig. 2A and 2B). Sciatic nerve clamp surgery induced peripheral nerve neuropathy. In addition, we observed coronal sections of the clamped sciatic nerve segment (Supplementary figure). Sciatic nerve clamping induced nerve injury with demyelination.

**Fig. 2:**
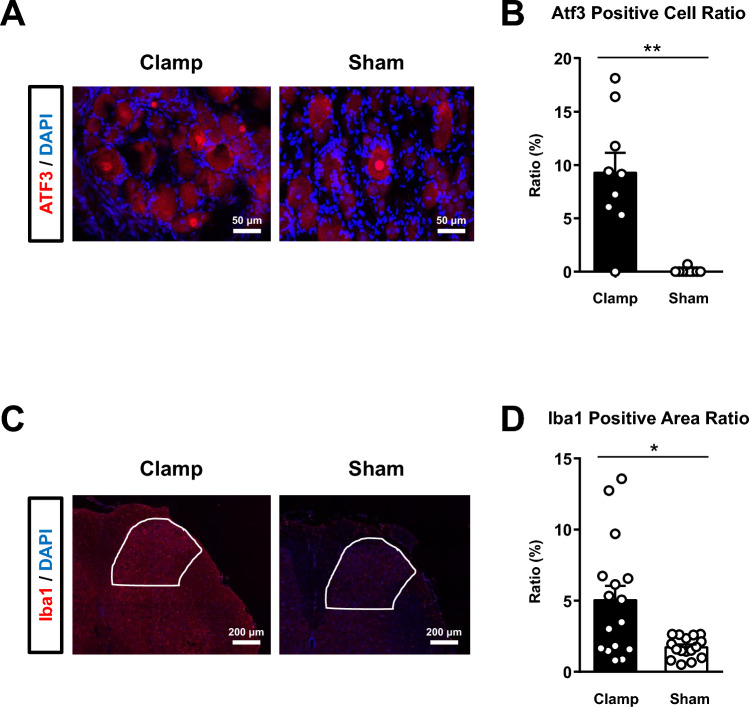
Histological analyses of the L5 dorsal root ganglion (DRG) and the spinal dorsal horn (SDH) after sciatic nerve clamp surgery. **A**. Representative immunofluorescence in the DRG of the rats that received sciatic nerve clamp surgery (Clamp, n = 3) and those that received sham surgery (Sham, n = 3). **B**. Bee swarm plot representing the ratio of Atf3 (red)-positive cells to control cells. The bars represent the means and standard errors of the means (SEM) in the two rat groups (3 sections from each rat). The ratio of Atf3-positive cells in the Clamp group was greater than that in the Sham group (9.3 ± 1.9% vs. 0.1 ± 0.1%, Mann–Whitney *U* test, **p < 0.01). **C**. Representative immunofluorescence in the SDH of Clamp and Sham rats (n = 4 rats each). The areas delineated by the white lines represent the SDH area of interest. **D**. Bee swarm plot representing the Iba-1 (red)-positive area ratio. The bars represent the means and standard errors of means (SEM) in the two groups (4 sections from each rat). The ratio of Iba-1-positive areas in the Clamp group was greater than that in the Sham group (5.0 ± 1.0% vs. 1.7 ± 0.2%, Mann–Whitney *U* test, *p < 0.05)

To evaluate the molecular changes to the spinal dorsal horn (SDH) induced by central sensitization after clamping of the sciatic nerve, we prepared 4 coronal sections of the SDH in the same manner as described above 14 days after surgery and stained them with anti-Iba1 IgG and DAPI. The ratio of the area of Iba1-immunopositive cells (i.e., microglial activation and proliferation) [24] to the area of the ipsilateral SDH was calculated using ImageJ software (National Institutes of Health, Bethesda, MD, USA; Fig. 2C and 2D). Sciatic nerve clamp surgery induced microglial activation and proliferation and elicited central sensitization in the SDH. Details of the methods are presented in the supplementary file.

The newly developed model of peripheral neuropathy induced by sciatic nerve injury causes both peripheral and central sensitization, resulting in mechanical allodynia, thermal hyperalgesia, and abnormal locomotion. Duloxetine attenuated mechanical allodynia and thermal hyperalgesia, suggesting that the sciatic nerve clamp model mimics human neuropathic pain. The model requires that the sciatic nerve is clamped for 10 min, and a shorter clamp time results in weaker responses. For example, George et al. previously reported that clamping the sciatic nerve for only 5 s resulted in neuropathy; however, allodynia did not occur, and only mild neuropathy was observed on day 10 [25, 26]. By contrast, longer clamping times may induce more severe pain. In our preliminary experiments, we used a von Frey test to evaluate rats whose nerves were clamped for 1, 5, and 10 min, and we adopted 10 min of clamping (data not shown). The clamping-induced pain-related behaviors returned to preoperative levels by day 28 (Fig. 1B and 1C). The duration of behavioral change was shorter than that in previous models [6–9, 11]. One of the advantages of our model is that the degree of neuropathy can be easily changed by altering the clamping time. In other words, compared with previous animal models, the intensity of pain can be easily changed. This is a useful feature of the present model; however, excessive neuropathy can cause irreversible sensory deficits and motor paralysis. Therefore, determining the optimal clamping time is critical for outcomes.

Our model was developed in the search for stem cell therapy for peripheral nerve injury or demyelinating diseases [27]. Experimental conditions, such as those used in this model, are essential for the development of cell transplantation therapies for neuropathic pain as the presence of foreign objects, such as surgical sutures around the transplanted stem cells, inhibits nerve regeneration [28, 29]. Our model may contribute to the development of innovative therapies for neuropathic pain.

We established an innovative rat model of neuropathic pain by clamping the sciatic nerve for 10 min. This new approach is notable for its ease of implementation and the consistency of the results.

## Supplementary Information

Below is the link to the electronic supplementary material.

Supplementary file1 (PPTX 135737 KB)

Supplementary file2 (DOCX 26 KB)

## Data Availability

The data are available from the corresponding author upon request.
