# Supplementary material for: Neuropathic pain after peripheral nerve injury in rats: a model using sciatic nerve clamping
Source: J Anesth. 2025 Aug 2;39(5):815–20. doi: 10.1007/s00540-025-03541-7 (PMC12464118; doi:10.1007/s00540-025-03541-7)
Supplement: Supplementary file 2 — Supplementary file2 (DOCX 26 KB) [file 540_2025_3541_MOESM2_ESM.docx]

**Materials and Methods (Supplementary File)**

*Animals*

We used a total of 46 male Wistar rats (8 weeks old, weighing 170–220 g). Only male rats were used to avoid the influence of gonadal hormone fluctuations associated with the 4-day estrous cycle of female rats on the observation of pain-related behavior during the 28-day period. All rats were housed for one week in an institutional animal husbandry area in groups of 3 or fewer in plastic cages on soft bedding at 23°C under a 12-hour light/dark cycle and allowed standard laboratory chow pellets and tap water ad libitum.

*Sciatic nerve clamp surgery*

The rats were transported from the institutional husbandry area to the laboratory. After at least 60 min of acclimation, general anesthesia was induced under 5% isoflurane anesthesia in 1 L/min of air administered from a vaporizer via a facemask. A longitudinal skin incision (approximately 3 cm) was made on the lateral side of the left thigh under 1.5% isoflurane anesthesia. The sciatic nerve was identified by blunt dissection. A disposable hemostatic clip (3.0 mm width, Natsume-Seisakusho, Tokyo, Japan) was used to clamp the nerve at an applied pressure of 60 g/mm^2^ for 10 min, 2 mm proximal to where the sciatic nerve trifurcates into peroneal, tibial, and sural nerves (Figure 1A). The clip was then declamped from the sciatic nerve, and the superficial muscle and skin were closed in each layer using 4-0 braided polyglactin 910 sutures (Vicryl; Ethicon, Bridgewater, NJ, USA) and 3-0 braided nylon sutures (Surgilon; Covidien, Minneapolis, MN, USA). In the sham-operated rats, the sciatic nerve was exposed in the same manner as described above but without clamping. The incisions were closed after 10 min.

*Behavioral testing*

We performed two somatosensory behavioral experiments (von Frey and Hargreaves tests) and one motor behavioral experiment (walking track test). All rats were allowed to acclimate for 20 min before the measurements were taken.

For the von Frey test, each rat was placed on a metal mesh floor in a small acrylic box (20 cm × 10 cm × 22 cm). The withdrawal response to non-noxious punctate mechanical stimulation was determined using calibrated monofilaments (von Frey filaments, Aesthesio Precision Tactile Sensory Evaluator, DanMic, San Jose, CA, USA) applied from beneath the mesh floor to the midplantar region of the left (ipsilateral) hind paw. Each filament was applied once, starting with 2.0 g of force, to a maximum force of 15 g, and a 50% withdrawal threshold was determined using the up–down method described by Dixon. Each rat was tested with at least a 5-minute interval between withdrawal responses. The von Frey test was repeated before and on days 1, 2, 4, 7, 14, 21, and 28 after surgery.

For the Hargreaves test, each rat was placed on a glass floor in a small plastic box (20 cm × 10 cm × 13 cm), and radiant heat from the lamp was projected onto the plantar surface of the hind paw using a Paw Stimulator Analgesia Meter (Model 390; IITC/Life Sciences Instruments, Woodland Hills, CA, USA). Hind paw withdrawal latency was measured to the nearest 0.1 s, and a cutoff of 20 s was used. Each rat was tested in the midplantar region of the hind paw 3 times, with at least 5 min between withdrawal responses. An average of 3 trials was used to calculate paw withdrawal latency after extreme outliers were excluded, such as repetitive unresponsiveness, which sometimes occurred when the plantar hind paw was wet with the rat’s urine. The Hargreaves test was repeated before and on days 1, 2, 4, 7, 14, 21, and 28 after surgery.

For the walking track test, the planter surfaces of the hind paws were painted with black ink. Each rat was placed on a sheet of white paper and guided to walk straight along a narrow alley toward a dark compartment. The footprint length between the 3rd toe and heel, the toe spread between the 1st and 5th toes of each hind paw, and the intermediate toe spread between the 2nd and 4th toes of each hind paw were measured in the most legible footprint. The sciatic functional index was calculated as described previously. A value of 0 indicates normal function, and a value of −100 indicates total impairment. The walking track test was performed before and on days 1 and 7, 14, 21, and 28 (once per week) after surgery to assess locomotor function recovery.

*Analgesic administration*

We administered diclofenac (10 mg/kg; Fujifilm Wako, Osaka, Japan), gabapentin (50 mg/kg; Fujifilm Wako), duloxetine (10 mg/kg; Fujifilm Wako), and 1 mL of normal saline as vehicle control intraperitoneally at 14 days after surgery (n = 5 in each group). We performed the von Frey and Hargreaves tests 30 min after drug administration.

*Immunofluorescence*

We performed histological analyses of the L5 dorsal root ganglia (DRG), spinal dorsal horn (SDH), and ipsilateral (left) and contralateral (right) sciatic nerves. The rats were deeply anesthetized with isoflurane and perfused transcardially with phosphate-buffered saline, followed by 4% paraformaldehyde. The target tissues were resected and immersed overnight in 4% paraformaldehyde on day 7 (DRG and nerves) or day 14 (SDH). After cryoprotection in sucrose, they were embedded in optimal cutting temperature compound (Tissue-TEK OCT compound; Sakura Finetek, Tokyo, Japan) and cut on a cryostat microtome into 10 μm-thick sections.

For immunostaining of the DRG, 6 rats were assigned to the clamp surgery group (n = 3) or the sham surgery group (n = 3). Three cross-sections from each DRG tissue sample were incubated overnight at 4°C with anti-activating transcription factor 3 (Atf3) IgG (1:200; HPA001562, Atlas Antibodies, Stockholm, Sweden). The sections were washed and incubated with secondary anti-rabbit IgG antibodies (1:500; A11011, Alexa568-conjugated; Thermo Fisher Scientific, Waltham, MA, USA). Nuclei were stained with DAPI solution (BS04; Dojindo, Kumamoto). Images of the sections were acquired using a confocal laser microscope (C2si Nikon Confocal Microscope System, Tokyo, Japan). The number of Atf3-positive cells was counted manually, and the ratio of positive cells to total cells was calculated.

For immunostaining of the SDH, 8 rats were assigned to the clamp surgery group (n = 4) or the sham surgery group (n = 4). Four cross-sections from each SDH tissue sample were incubated overnight at 4°C with primary anti-Iba1 IgG (1:200; #019-19741; Fujifilm Wako). The sections were washed and incubated with secondary anti-rabbit IgG antibodies (1:500; A11011, Alexa568-conjugated; Thermo Fisher Scientific). Nuclei were stained with DAPI solution (BS04; Dojindo). Images of the sections were acquired using a confocal laser microscope (C2si Nikon Confocal Microscope System). The area of the SDH was delimited medially, dorsally, and laterally by the white matter–gray matter border and ventrally by a horizontal line originating at the level of the inflection point, where the lateral white matter–gray matter border curves the most inward. The ratio of the area of Iba1-positive cells to the area of the ipsilateral SDH was calculated using ImageJ software (ver. 1.54g, National Institutes of Health, Bethesda, MD, USA).

For immunostaining of the bilateral sciatic nerves, cross-sections of the middle portion of each nerve were incubated overnight at 4°C with anti-neurofilament IgG (1:200; AB1987, Merck Millipore, Burlington, MA, USA) and anti-myelin basic protein (MBP) IgG (1:500; SMI-99P, BioLegend, San Diego, CA, USA). The sections were washed and incubated with Alexa568-conjugated secondary anti-rabbit or anti-mouse IgG antibodies (1:500; A11011/A10680; Thermo Fisher Scientific, Waltham, MA, USA). Nuclei were stained with DAPI (BS04; Dojindo). Images of the immunofluorescence-stained sections were acquired using a confocal laser microscope (C2si Nikon Confocal Microscope System).

*Statistical analysis*

Unless otherwise noted, all data are presented as the means ± standard errors of the means (SEM). All statistical analyses were performed with Prism (GraphPad Software, La Jolla, CA, USA). Differences in continuous variables were tested using a two-way repeated measures ANOVA followed by a Sidak test, Kruskal–Wallis test followed by a Dunn test, or a Mann–Whitney *U* test. Differences with *p* < 0.05 were considered significant.
